# Photooxidation Tolerance Characters of a New Purple Pepper

**DOI:** 10.1371/journal.pone.0063593

**Published:** 2013-05-21

**Authors:** Li-jun Ou, Zhu-qing Zhang, Xiong-ze Dai, Xue-xiao Zou

**Affiliations:** 1 Vegetable Institution of Hunan Academy of Agricultural Science, Hunan Changsha, China; 2 Key Laboratory of Hunan Province for Study and Utilization of Ethnic Medicinal Plant Resources, Huaihua University, Hunan Huaihua, China; 3 Department of Life Sciences, Huaihua University, Hunan Huaihua, China; University of East Anglia, United Kingdom

## Abstract

Huai Zi (HZ) is a new purple mutant of green pepper (PI 631133) that is obtained from the United States Department of Agriculture. The net photosynthetic rate (*P*_N_), chlorophyll fluorescence parameters, antioxidant substances, antioxidant enzymes, photosystem 1 (PS1) and PS2 activities were studied through methyl viologen (MV) treatment. The results showed that the *P*_N_, actual photochemical efficiency of PS2 (ΦPS2), photochemical quenching coefficient (q_P_), PS1 and PS2 activities in HZ were lower than those in green pepper. HZ had a stronger ability to eliminate reactive oxygen species(O_2_^•−^) and accumulated less malondialdehyde (MDA) (a membrane lipid peroxidation product) than did green pepper, and had a higher content of antioxidants and antioxidant enzyme activity. This suggests that the lower light energy absorption and higher thermal dissipation and antioxidant activity of HZ contributed to a more stable PS2 photosynthetic capacity, which resulted in photooxidation tolerance. Hence, our study strongly suggests that pepper hybrids can achieve a modest ratio of chlorophyll and anthocyanin content, high *P*_N_ and resistance to photooxidation, improving yield and resistance to adverse environments.

## Introduction

Plants might experience photooxidation under natural conditions [1], [2], [3], directly limiting photosynthetic productivity [4], [5] and resulting in decreased yields. The photosynthetic organs have developed photoprotective mechanisms to minimize potential damage caused by light. These mechanisms include the reduction of the direct absorption of light energy by changes in the blade angle, chloroplast movements and waxy layer thickness, protection through state transitions, cyclic electron transport, D1 protein turnover, antioxidant molecules and enzyme systems, and consumption of heat energy through non-radiative dissipation [6]. The first defence mechanism consists of the physiological characteristics that confer long-term protection, while the second line of defense is the immediate photoinhibition responses that provide effective protection in the short term. The synergies of the different protection mechanisms reduce the damage caused by photoinhibition. Therefore, studying the physiological basis of tolerance to high irradiance is an important strategy for obtaining stable, high yielding varieties.

Green pepper has poor tolerance to high temperature and strong light [7], [8]. Thermophilic vegetable production often happens when sudden stresses such as high temperature or strong light at noon generate new resistant varieties [9]. Purple pepper is a rare pepper [10] that owes its colour to anthocyanin synthesis and accumulation. Some studies have shown that purple pepper has good resistance to stress and disease, and at the same time, the purple colour serves as an early trait marker in pepper hybrids to improve the efficiency of breeding selection [11], [12], [13]. Anthocyanins have significant antioxidant effects [14], [15]. Purple peppers are mainly selected by artificial breeding [16], [17]. HZ is a natural purple mutant that was selected from green pepper; its most important characteristic is its permanent purple colour. In this paper, the purple pepper mutant was used to study photooxidation tolerance by measuring the photosynthetic characteristics, enzymatic activity and photooxidation products. This study provides a theoretical basis to select varieties with high photosynthetic efficiency and suitable for cultivation under high temperature and strong summer irradiance conditions.

## Results

### Pigments

HZ had 10.4 times the anthocyanin content of green pepper. Under normal conditions, the contents of chlorophyll (Chl) a and b and carotene (Car) in HZ were slightly lower than those in green pepper, while the content of Chl a/b was higher than that of the control. Under photooxidation stress, the contents of photosynthetic pigments and Chl a/b in the peppers decreased, but HZ experienced a significantly lower decrease than that observed in green pepper (Table 1).

**Table 1:** Pigment content [g kg^–1^(DM)] in pepper.Chl – chlorophyll.

pigment	Chl *a*			Chl *b*			Chl *a/b*		
	Control	Treatment	–%	Control	Treatment	–%	Control	Treatment	–%
HZ	8.93±0.58	5.45±0.24	38.97*	2.41±0.11	1.54±0.14	36.01**	3.71	3.55	4.31**
WT	9.31±0.42	4.78±0.36	48.66	2.89±0.23	0.92±0.09	68.17	3.22	3.14	2.54

Note: HZ– the mutant of purple pepper; WT– the wild pepper of green pepper (PI631133). Values are means ± SE (*n* = 9).

** ,*t-test significance at 0.01and 0.05 levels, respectively.

### Gas Exchange

**Figure 1:**
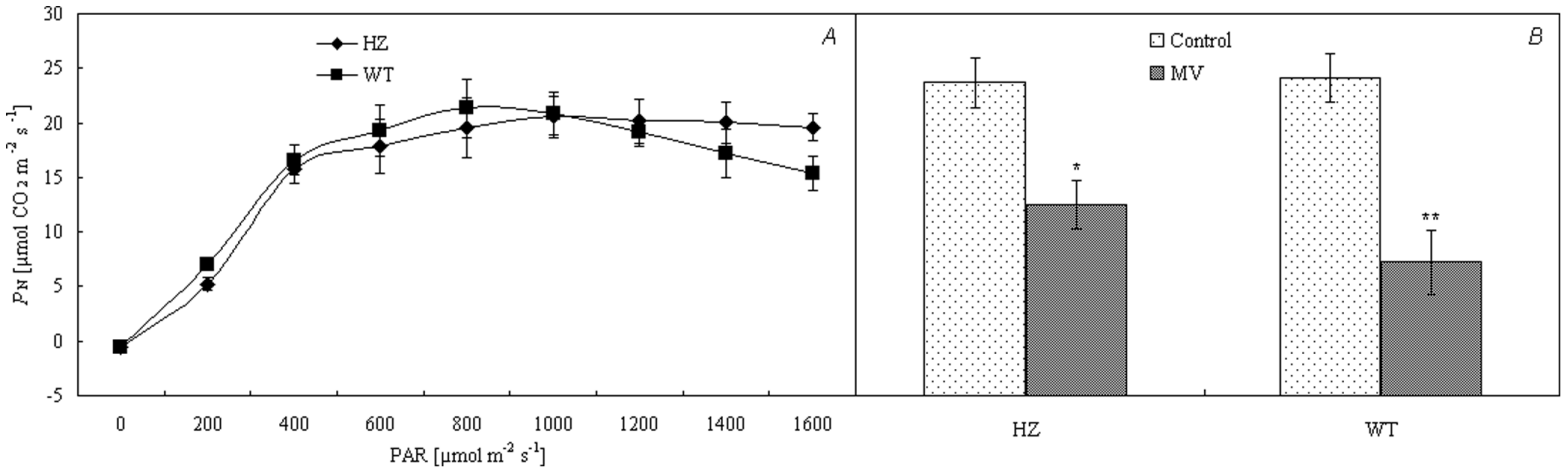
Photosynthetic rate (*P*_N_) under different irradiances (A) and treatment with MV (B) of different pepper. **,*t-test significance at 0.01and 0.05 levels, respectively. Note: HZ– the mutant of purple pepper; WT– the wild pepper of green pepper (PI631133); MV – methyl viologen. Values are means ± SE (*n* = 9).

The *P*_N_ of the peppers tended to increase and peaked with irradiance strength. The highest *P*_N_ of HZ was higher than that of green pepper under normal conditions (Fig. 1*A* ). Under photooxidation treatment, *P*_N_ decreased, but the decrease seen in HZ was lower than that seen in the wild type (Fig. 1*B*). The light compensation point and light saturation point of HZ were significantly higher than those of green pepper, while its apparent quantum yield was significantly lower than that of green pepper (Table 2).

**Table 2:** Light compensation point(LCP), light saturation point(LSP), photosynthetic rate at light saturation point(PR_LS_) and apparent quantum yield(AQY)of pepper, ^**,*^ *t*-test significance at 0.01and 0.05 levels, respectively.

	LCP/µmol•m^−2^•s^−1^	LSP/µmol•m^−2^•s^−1^	PR_LS_/µmol•m^−2^•s^−1^	AQY/mol•mol^−1^
HZ	21.83±0.58**	1112.48±21.12*	28.01±4.11*	0.0566±0.0009**
WT	11.31±0.42	947.45±11.21	21.09±3.23	0.1096±0.0011

Note: HZ– the mutant of purple pepper; WT– the wild pepper of green pepper (PI631133). Values are means ± SE (*n* = 9).

### Chl Fluorescence

Fluorescence analysis of chlorophyll showed that photooxidation stress had significant impact on PS2. optimal/maximal photochemical efficiency of PSII in the dark (Fv/Fm), quantum efficiency of PSII photochemistry (ΦPS2) and photochemical quenching coefficient (q_P_) decreased significantly. The decreases of Fv/Fm, q_P_ and ΦPS2 in HZ were 11.75%, 27.27% and 37.68%, respectively, and were significantly lower than the decreases observed in green pepper (Table 3).

**Table 3:** Chlorophyll fluorescence kinetic after photooxidation treatment.

pigment	Fv/Fm			qP			ΦPSII		
	Control	Treatment	–%	Control	Treatment	–%	Control	Treatment	–%
HZ	0.825±0.07	0.728±0.08	11.75**	0.583±0.01	0.424±0.01	27.27**	0.406±0.006	0.253±0.006	37.68**
WT	0.831±0.07	0.610±0.06	26.59	0.608±0.01	0.360±0.01	40.78	0.443±0.007	0.214±0.004	51.69

Note: HZ– the mutant of purple pepper; WT– the wild pepper of green pepper (PI631133);

Fv/Fm - optimal/maximal photochemical efficiency of PSII in the dark; q_P_ – photochemical; ФPS2– actual photochemical efficiency of PS2quenching coefficient. Values are means ± SE (*n* = 9).

** ,*t-test significance at 0.01and 0.05 levels, respectively.

### Photooxidation Products

**Figure 2:**
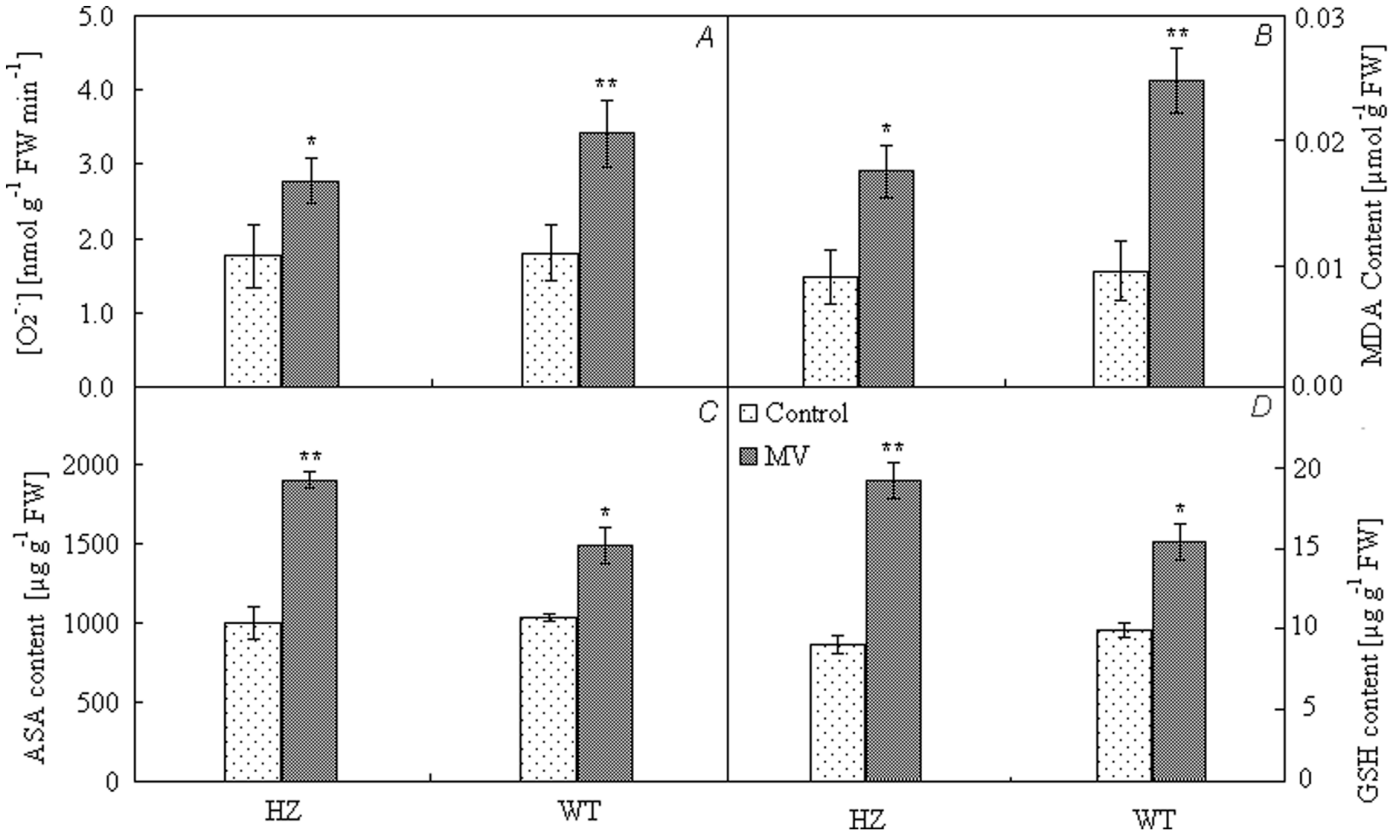
Content of photooxidation and antioxidation products after photooxidation treatment. **,*t-test significance at 0.01and 0.05 levels, respectively. Note: HZ– the mutant of purple pepper; WT– the wild pepper of green pepper (PI631133); O_2_^•−^ – reactive oxygen species; MDA – malondialdehyde;ASA – Ascorbic acid; GSH – glutathione. Values are means ± SE (*n* = 9).

The production rate of O_2_^•−^ and the content of MDA significantly increased after photooxidation treatment (Fig. 2*A,B*). For HZ, the increases in the production rate of O_2_^•−^ and the content of MDA were 57.46% and 93.76%, respectively, while for green pepper they were 89.28% and 159.26%, respectively, indicating that the clearance rate of photooxidation products in purple pepper was larger than that in in green pepper.

### Antioxidant Products

Ascorbate- glutathione (AsA-GSH) cycle is one way to eliminate photooxidation products. The contents of ASA and GSH in HZ increased after photooxidation treatment (Fig. 2*C,D*)). The increases of ASA and GSH in HZ were 89.88% and 112.61%, respectively, while for green pepper they were 43.50% and 54.51%, respectively.

### Antioxidant Enzyme Activity

**Figure 3:**
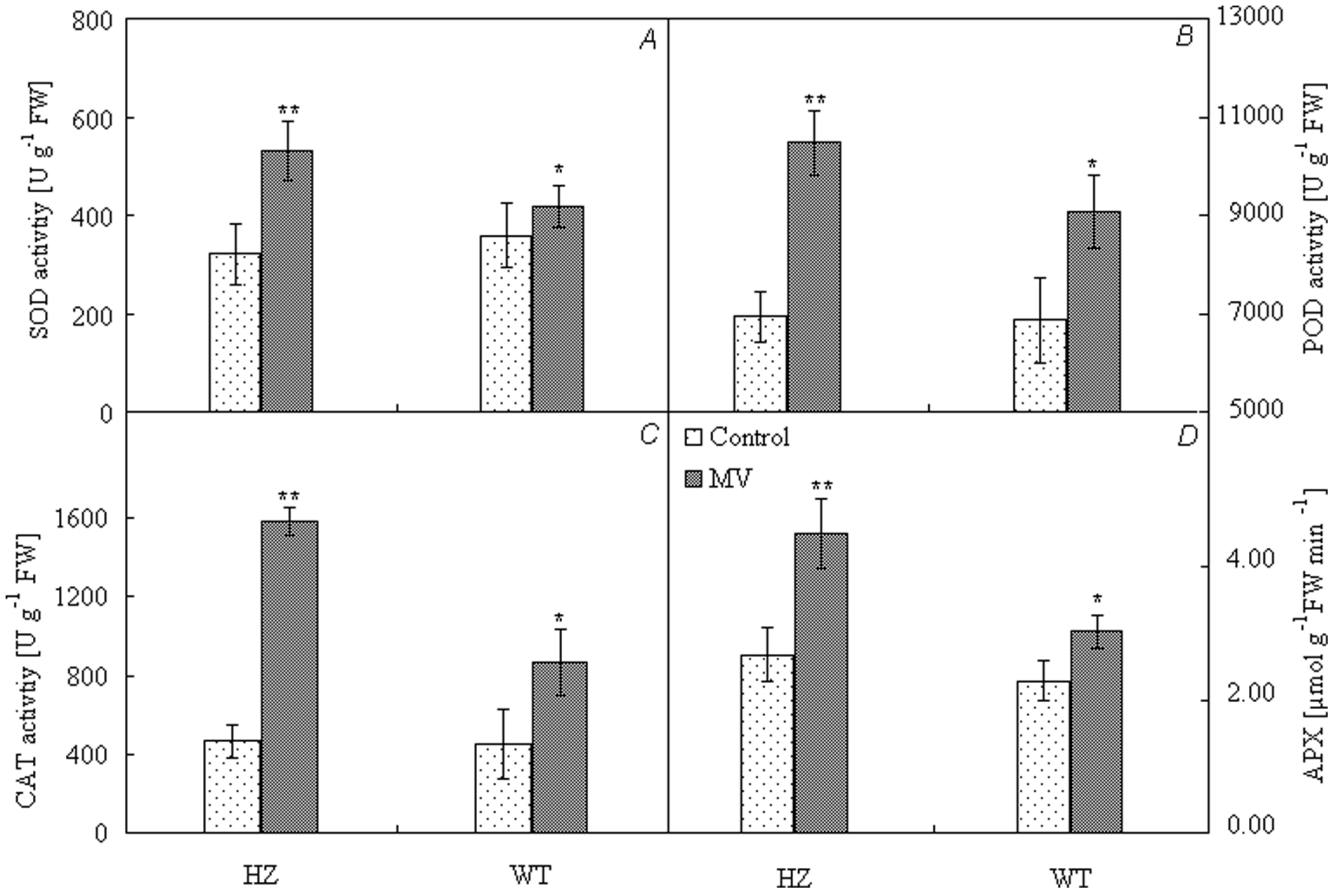
Enzyme activity of antioxidation after photooxidation treatment. **,*t-test significance at 0.01and 0.05 levels, respectively. Note: HZ– the mutant of purple pepper; WT– the wild pepper of green pepper (PI631133); SOD – Superoxide dismutase; CAT – catalase; APX – ascorbate peroxidase; POD – guaiacol-dependent peroxidase. Values are means ± SE (*n* = 9).

Under long-time photooxidation conditions, superoxide dismutase (SOD), CAT –; peroxidase(POD), catalase (CAT) and ascorbate peroxidase (APX) activities were higher and of longer duration, while the generation and accumulation of O_2_^•−^ was lower in the tolerant varieties. Under photooxidation stress, the SOD, POD, CAT and APX activities of pepper increased. SOD, POD, CAT and APX activities in HZ increased by 65.42%, 51.17%, 239.73% and 68.04%, respectively, and were significantly higher than those of green pepper (Fig. 3).

### PS1 and PS2 Activities

**Figure 4:**
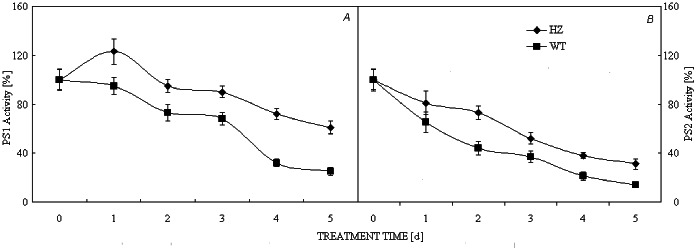
PS1 and PS2 activities after photooxidation treatment. **,*t-test significance at 0.01and 0.05 levels, respectively. Note: HZ– the mutant of purple pepper; WT– the wild pepper of green pepper (PI631133). Values are means ± SE (*n* = 9).

These activities were also used to measure the antioxidant capacity during the photooxidation treatment. PS1 activity in HZ increased during the first day of treatment and decreased progressively thereafter. On the other hand, PS1 activity in green pepper decreased from the start. Furthermore, the degree of decrease was always greater in green pepper than in HZ (Fig. 4*A*). In contrast, PS2 activity decreased immediately after treatment and was higher in HZ than in green pepper (Fig. 4*B*). Comparison of the rates of change between PS1 and PS2 showed that PS2 is more prone to photooxidative damage.

## Discussion

Chloroplasts are the main photosynthetic apparatus and the most sensitive organelles to photooxidation. Under normal conditions, the light energies absorbed by chloroplasts are mainly consumed through the photosynthetic electron transport, chlorophyll fluorescence and heat dissipation. These three pathways compensate each other; thus, fluorescence changes can reflect the photosynthesis status [18], [19], [20]. The original damage site of photosynthesis is closely linked with the PS2 system. Under excessively high irradiance, the primary site of photooxidation may be located in the PS2 reaction centre, and related to the rapid turnover of the D1 protein under radiation stress, which leads to its depletion and the loss of PS2 activity [21], [22], [23], [24], [25]. In this experiment, we found that F_v_/F_m_ and ΦPS2 of PS2 significantly decreased in pepper under photooxidation treatment, indicating that the primary conversion efficiency of light energy and potential activity of PS2 were inhibited, directly affecting the photosynthetic electron transport and CO_2_ assimilation. Meanwhile, the increased non-photochemical quenching q_N_ indicated that pepper was able consume excess light energy through heat dissipation, avoiding damage to the photosynthetic apparatus. This might be a protective mechanism used by pepper to adapt to its environment. Our results also showed that the decrease in Fv/Fm and qP in HZ was significantly lower than that observed in green pepper, indicating that purple pepper had a higher tolerance to photooxidation. Previous studies demonstrated that the AsA-GSH cycle is one of the major systems used to eliminate H_2_O_2_ [26]. SOD, CAT, APX [6], [27] and PS1 and PS2 activities activities [21] have antioxidant effects and contribute to plant protection. Our results showed that HZ had significantly higher activities of SOD, CAT, POD and APX enzymes, and less marked decreases of PS1 and PS2 activities than those seen in green pepper. HZ had significantly lower contents of MDA and O_2_^•−^, suggesting that it might be able to effectively protect chloroplasts and express a higher tolerance against photooxidation.

Purple pepper owes its colour to anthocyanins from the leaves. Anthocyanins showed an absorption peak at 540 nm in the visible region, indicating that purple leaves absorb few low energy rays, and another absorption peak at 265 nm in the ultraviolet region, suggesting injury resistance against high irradiance or inactivation of enzymes to a certain extent [28]. Our study showed that, under normal conditions, q_P,_ andΦPSII in HZ were lower than in green pepper; however, they were higher under photooxidation treatment, indicating that HZ was able to use the captured energy efficiently. On the other hand, purple pepper did not have a lower chlorophyll content, although it had more anthocyanins, and differences in the photosynthetic capacity were not due to the chlorophyll content. This was proved in purple-leaf plum [29] and purple cabbage [30]. Usually, high temperature is the primary impact factor towards photosynthesis in summer, and strong light often acts together with high temperature to affect the speed of the photochemical reaction and the activity of the photosynthetic enzymes [31], which induce changes in the structure and function of PS2 [32]. Pepper often suffers from high irradiance exposure during nutrition and reproductive growth, and the leaves experience severe photooxidative damage. This study suggests that we should use hybridization to select peppers with a modest ratio of chlorophyll to anthocyanin content, as well as high *P*_N_ and resistance to photooxidation, to achieve high photosynthetic efficiency in high temperature, strong light and low humidity conditions.

## Materials and Methods

### Plants

HZ, a purple mutant of green pepper (PI 631133), was obtained from the United States Department of Agriculture. Wild type pepper was used as a control. The plants were grown using standard amounts of fertilizer and water.

### Photooxidative Treatment

The method of Lin et al. [33] was adopted to generate photooxidative stress in leaves in mid-August (flowering stage). The upper surface of intact leaves was smeared with an oxidative reagent containing 1.5 mM MV and 1% v/v tween-80 for five consecutive days. Distilled water containing 1% v/v tween-80 was used as a control.

### Pigment Analysis

Anthocyanin content was measured according to Sui et al. [28] and contents of Chl a and b, carotenoids and Car were determined by the method of Arnon [34].

### Gas exchange

The method of Yong et al. [35] was determined them during 09∶00−11∶00 (Beijing time) in mid-August (flowering stage) using Li-6400 (*Li-Cor*, USA). For determining the response to irradiance, *P*_N_ was measured at photosynthetic photon flux density of 2 000, 1 800, 1 600, 1 400, 1 200, 1 000, 800, 600, 400, 200, 100, 50, 25, and 0 µmol·m^−2^·s^−1^ in turn from a *Li-Cor* LED irradiation source. CO_2_ concentration was kept at 400 µmol·mol^−1^ with *Li-Cor* CO_2_ injection system. Leaf temperature was 30°C. LCP, LSP and AQY were determined by the method of Sui et al. [36].

### Chl Fluorescence Kinetics

They were measured according to Li [37]: The minimal initial fluorescence (F_o_) and maximal fluorescence (F_m_) were determined after dark adaptation for 20 min. Then minimal fluorescence (F_o_’) and maximal fluorescence under light (F_m_’) were measured after a 1-h irradiation. Other fluorescence parameters were calculated according to Genty *et al*. (1989): q_P_ = (F_m_’ − F_s_)/(F_m_’ − F_o_’); q_N_ = (F_m_ − F_m_’)/(F_m_ − F_o_); Φ_PS2_ =  (F_m_’ − F_s_)/F_m_’. All these parameters were measured using *Li 6400*.

### ASA and GSH Measurements

Leaves were ground in 3 mL of 5% trichloroacetic acid (TCA) and centrifuged for 10 min at 15,500*×g*. The supernatant was adjusted to 3 mL. ASA was assayed according to Tanaka et al. [38]. In brief, 0.8 mL of 150 mM NaH_2_PO_4_ (pH 7.4) was added to 0.4 mL of sample, and 0.8 mL of 10% trichloroacetic acid, 0.8 mL of 44% phosphate, 0.8 mL of 4% bipyridine and 0.4 mL of 3% FeCl_3_ were added to the solution. After incubating at 37°C for 1 h, ASA was determined by reading the absorbance at 265 nm, based on the standard curve. GSH was assayed according to Ellman [39]. In brief, 2.6 mL of 150 mM NaH_2_PO_4_ (pH 7.7) and 1.2 mL of 5,5-dithio-bis(2-nitrobenzoic acid) were added to a 0.2 mL sample. After incubating at 30°C for 5 min, GHS was determined by reading the absorbance at 412 nm, based on the standard curve obtained using phosphate buffer instead of 5,5-dithio-bis(2-nitrobenzoic acid) as a control.

### Assays of Enzyme Activities

The enzyme activities were assayed according to García-Limones et al. [40]. In brief, 1 g of vein-free leaves was homogenized in ice-cold 50 mmol/L phosphate buffer (pH 7.8) containing 1.0 mmol/L ethylene diamine tetraacetic acid. The homogenate was centrifuged at 15,000*×g* for 10 min at 4°C. The supernatant was used to measure the activities. APX activities were determined in the presence of 0.5 mmol/L ascorbic acid and 0.5 mmol/L H_2_O_2_ by monitoring the decrease in absorbance at 290 nm. CAT activities were determined spectrophotometrically by monitoring the decrease in absorbance at 240 nm. SOD activities were assayed by measuring the inhibition of the photochemical reduction of nitro-blue tetrazolium. Analysis of guaiacol POD capacity was based on the oxidation of guaiacol, using hydrogen peroxide. The reaction mixture contained 2.5 mL of 50 mmol/L potassium phosphate buffer (pH 6.1), 1 mL of 1% hydrogen peroxide, 1 mL of 1% guaiacol and 10–20 µL of enzyme extract. The increase in absorbance was read at 420 nm.

### Determination of O_2_^•−^

The method of Wang and Luo [41] was applied. Leaf segments (about 5 g fresh mass) were homogenized using a cold pestle and mortar with acid-washed quartz sand in 65 mM phosphate buffer (pH 7.8). The homogenate was filtered through four layers of gauze. The filtrate was centrifuged at 5000*×g* for 10 min at 4°C. Then, 0.9 mL of phosphate buffer and 0.1 mL of 10 mM hydroxylamine hydrochloride were added to the supernatant. The mixture was incubated at 25°C for 20 min. Then, 0.5 mL of the incubated mixture was added to 0.5 mL of 17 mM sulfanilic acid, and kept at 25°C for 20 min. The developing solution was shaken with an equal volume of n-butanol and subsequently separated into two phases. The n-butanol phase was taken out and measured at 530 nm. Phosphate buffer without sample was used as a control. For samples containing large amounts of chlorophyll, ethyl ether replaced n-butanol and the mixture was centrifuged at 1500*×g* for 5 min. The absorbance of the water phase was measured at 530 nm. O_2_^•−^ production was calculated from the standard curve of the developing NO_2_^−^ reaction, using the formula: O_2_^•−^ production rate =  O_2_^•−^ production/reaction time × amount of protein [mmol(O_2_^•−^) mg^−1^(protein) min^−1^].

### MDA Content

The content of MDA was determined using the method described by Hodges et al. [42]. In brief, 0.5 g of vein-free leaves were ground in 5 mL of 10% trichloroacetic acid solution. The homogenate was centrifuged at 4000*×g* for 10 min and 2 mL of the supernatant (2 mL H_2_O as a control) was mixed with 2 mL of 0.6% thiobarbituric acid. The mixture was heated at 100°C for 15 min, cooled and then centrifuged at 10,000*×g* for 5 min. The absorbance was recorded at 600, 532 and 450 nm and the content of MDA (µmol g^−1^) was calculated with the following formula: [MDA (µmol L^−1^) ] = 6.45(A_532_–A_600_)–0.56A_450_.

### PS2 Activity

The PS2 activity of isolated thylakoids was measured with a polarographic oxygen electrode, as described by Shyam [43]. The reaction mixture contained 50 mM Tricine-KOH (pH 7.0), 5 mM MgC1_2_, 1 mM K_3_Fe(CN)_6_, 10 µM 2,5-dibromo-3 -methyl-6-isopropyl β-benzoquinone, and chloroplast suspension at a final concentration of 5 g Chl m^−3^.

### PS1 Activity

The isolation of intact chloroplasts and measurement of PS1 activity with an oxygen electrode were described previously. The reaction mixture contained 50 mM Tris-HC1 (pH 7.8), 10 mM NaCl, 50 mM NH_4_Cl, 20 µM 2,6-Dichlorophenolindophenolsodium salt, 50 µM MV, 10 µM 3-3,4-dichlorophenyl-1,1-dimethylurea, and chloroplast suspension equivalent to 50 mg of Chl.

### Statistical Analysis

Significances were tested by one-way or two-way ANOVA, and the results are expressed as the mean values ± SD of nine independent experiments.
